# An audit on the assessment and management of osteoporosis in a Parkinson’s and related diseases clinic in Australia

**DOI:** 10.1007/s00415-024-12752-z

**Published:** 2025-01-15

**Authors:** Nethmi Nuwanji Amarasekera, Janice Taylor, Christopher Coppin, Simon J. G. Lewis

**Affiliations:** 1 Imperial College School of Medicine, Imperial College London, London, UK; 2 Macquarie Medical School, Parkinson’s Disease Research Clinic, Macquarie University, Sydney, NSW 2109 Australia

**Keywords:** Parkinson’s disease, Falls, Osteoporosis, Bone health, Minimal trauma fracture

## Abstract

**Background:**

Patients with Parkinson’s disease (PD) and atypical parkinsonian syndromes are at increased risk of falls and should be actively screened and treated for osteoporosis. In 2024, the Royal Australian College of General Practitioners (RACGP) revised their practice guidelines for diagnosing and managing osteoporosis in postmenopausal women and men aged over 50 years.

**Objective:**

We conducted the first Australian study to audit these guidelines in patients with PD and atypical parkinsonian syndromes.

**Method:**

We audited all PD, Dementia with Lewy Bodies, Progressive Supranuclear Palsy and Multiple System Atrophy cases attending our neurology service between January and March 2024 against the RACGP osteoporosis guidelines. We identified patients at risk of osteoporosis or minimal trauma fractures and assessed if they had been referred to their general practitioner (GP) for appropriate management or were already receiving appropriate osteoporosis treatment.

**Results:**

This audit evaluated 230 patients, 199 of which had PD. We identified 78 patients over the age of 50 years with risk factors that should trigger a GP bone health assessment as per the guidelines. Twenty-six of these patients were already being managed appropriately. However, only 12 of the remaining 52 ‘at risk’ patients (23%) were directed to seek screening for osteoporosis by their GP, leaving 77% (40/52) without appropriate guidance.

**Conclusion:**

Our major recommendations include following the guidelines and referring patients for a bone health screen with their GP if they have risk factors for osteoporosis. This audit highlighted that assessment of osteoporosis and fracture risk by Specialists needs to be improved.

**Supplementary Information:**

The online version contains supplementary material available at 10.1007/s00415-024-12752-z.

## Introduction

Osteoporosis is characterised by reduced bone tissue microarchitecture and bone mineral density (BMD) [1]. This pathology significantly increases bone fragility, leading to fractures that are typically triggered by minimal trauma, such as falls from a standing height or less. These fractures are associated with substantial morbidity and mortality in elderly patients [1]. It is well known that individuals with Parkinson's Disease (PD) are over twice as likely to develop osteoporosis and sustain fractures [2]. This may be due to reduced sunlight exposure, vitamin D deficiency, disease duration and severity, immobility, and low body mass index [3]. Patients with PD also have an increased risk of fractures from falls due to postural instability, motor fluctuations, freezing of gait and physical deconditioning [4]. Osteoporotic fractures observed in PD are associated with greater costs and morbidity than fractures related to other conditions [4]. Despite the well-established association between osteoporosis and female gender, men with PD sustain almost as many hip fractures as women with PD, underlining that bone health assessment is equally important for men and women [5]. The atypical parkinsonian syndromes including Dementia with Lewy bodies (DLB), Multiple System Atrophy (MSA) and Progressive Supranuclear Palsy (PSP) are all characterised by an increased risk of falls and are likely to be seen by the same specialists as patients with PD, typically a Neurologist or Geriatrician [6].

Individuals who are at increased risk of minimal trauma fractures should be screened to guide diagnosis and therapies for osteoporosis [1]. Screening is completed using dual energy X-ray absorptiometry (DEXA) scans that measure BMD [1]. However, fracture risk assessment tools should be used prior to DEXA imaging to reduce the number of patients being unnecessarily scanned [1]. Whilst there has been much debate around which fracture risk assessment tools are most appropriate [2], the Fracture Risk Assessment Tool FRAX® [available at https://fraxplus.org] is currently the most widely used, with multiple versions available depending on the country of use. The FRAX provides a probability of major osteoporotic fracture (MOF) in the next 10 years, which helps determine the necessity of conducting a DEXA scan. It is currently the only tool to incorporate previous DEXA scores, where available, to calculate future risk. It is an online tool where risk factors are selected if present and the alternative QFracture tool is based on a UK-based open cohort study [7] and has previously been recommended specifically for use relating to PD [2]. Whilst the FRAX provides 10-year probability of MOF, QFracture provides a 1–10 year cumulative probability, increasing its utility in patients who have a lower life expectancy, as can be the case in patients with PD and atypical parkinsonian syndromes [2].

In 2019, a UK-wide clinical audit was conducted by the Parkinson’s Excellence Network [8]. It highlighted bone health as an area requiring improvement amongst patients with PD [8]. Following this, a service improvement project was undertaken across the UK involving 1131 people with Parkinson’s from 44 specialist services (elderly care and neurology) [9]. The project aimed to improve rates of assessing and treating osteoporosis by encouraging the multidisciplinary team to use FRAX or QFracture risk assessment tools to highlight those that may need a more detailed investigation into their bone health [10]. The project resulted in bone health assessment in neurology clinics across the UK improving by around 20% in the 2022 audit cycle by Parkinson’s Excellence Network [9]. Several smaller audits conducted in the UK have also demonstrated that screening and management of bone health in specialist PD neurology clinics is generally suboptimal [11, 12].

In March 2024, the Royal Australian College of General Practitioners (RACGP) updated their practice guidelines for diagnosing and managing osteoporosis [1], revising their previous advice published in 2017 [13]. The 2024 guidelines [1] first recommend that a general practitioner (GP) should assess fracture risk in all patients over the age of 50, identifying both non-modifiable and lifestyle risk factors using a tool such as FRAX. These risk factors include age over 70 years, history of multiple falls, poor balance, and immobility. The guidelines further suggest that patients over 50 years with a prior minimal trauma fracture, on certain medications (e.g. prolonged higher dose glucocorticoids, androgen deprivation therapy, aromatase inhibitors), or patients with certain diseases (e.g. rheumatoid arthritis, diabetes, coeliac disease) should have a DEXA scan to objectively evaluate their BMD every 1–2 years. Patients who have already sustained a minimal trauma fracture of the hip or vertebra have a presumptive diagnosis of osteoporosis and can be started on medication without a DEXA scan. The degree of osteopenia is also an important factor that should encourage closer monitoring of fracture risk [1]. The guidelines also make recommendations for general bone health. They state that all patients should be encouraged to exercise, ideally with resistance or weight bearing exercises. Frail or institutionalised individuals should have their vitamin D and calcium levels optimised, and their dietary intake and sun exposure should be monitored, with supplements provided if insufficient. There is insufficient evidence to recommend supplements to otherwise healthy individuals [1].

The current Australian guidelines do not specifically mention PD or how to incorporate the impact of the condition on screening for fracture risk [1]. Despite their predisposition for osteoporosis and minimal trauma fractures in PD patients, previous audits have shown that around half of these patients do not have their bone health assessed following their specialist clinic appointment [8, 9]. Typically, such patients are reviewed by Neurologists and Geriatricians [9], suggesting that an interdisciplinary and integrated approach is required to improve osteoporosis and fracture risk management in these patients. Therefore, we conducted an audit to raise awareness of the fracture risk amongst patients in our clinic and as a scoping exercise to develop local PD specific guidelines.

Our study was impacted by the Australian guidelines changing during the period covered by this audit. Our standards were derived primarily from the 2024 RACGP osteoporosis guidelines with some influence from the 2017 guidelines [1, 13]. In the 2017 RACGP guidelines, the indications for a fracture risk assessment or DEXA scan varied by the strength of evidence to support their implementation, whereas the 2024 guidelines presented all recommendations equally. We chose to mainly adopt the 2024 practice guidelines and their associated grade of evidence. However, since the 2024 practice guidelines did not provide the strength of evidence for certain recommendations (for example, referring all patients over 70 years for a FRAX score), we made a judgement based on the level of evidence indicated in the 2017 guidelines. Regardless of this subtle change in emphasis of the revised RACGP guidelines, we believe that identifying current practice in the specialist clinic setting should help to improve future practice and highlight the need to refer patients with a high likelihood of fragility fracture for osteoporosis screening with their GP.

### Method

We identified all PD, DLB, MSA and PSP cases that attended our neurology outpatient service between January and March 2024 using the established diagnostic criteria [14–18]. One member of the team (NA) audited the clinic letters for these cases against the existing osteoporosis RACGP guidelines [1]. Records were checked for age, sex, any current bone health diagnoses or medication, fracture and falls history, mobility, exercise, and past medical history. Bone health medication reviewed included bisphosphonates, denosumab, vitamin D and calcium supplements. There are many lifestyle factors that warrant a FRAX assessment according to the RACGP 2024 guidelines [13]. However, only the following factors were audited due to their prevalence in PD and relevance to the condition's pathology: falls, poor balance, and immobility [19]. Any disparities regarding clinic letter data, audit criteria or recommendations were discussed with other members of the team to obtain consistency. We aimed to identify patients at risk of osteoporosis or minimal trauma fractures and assess if they had been referred to their GP for appropriate management. The recommendations were developed following presentation and discussion of the audit results with other members of the team.

## Results

This audit evaluated 230 patients who were reviewed by 5 healthcare professionals, including doctors and nurses, at 1 centre. Whilst the RACGP guidelines do not specify management for those under 50 years, we identified seven such patients (Table 2), of whom three likely should have been investigated further based on increased risk of osteoporosis due to falls (*n* = 1) or history of prior fracture (*n* = 2). One of these patients had a pelvic fracture on a background of poor balance and a 7-year history of PD. Despite being under 50 years, this patient should probably have been referred due to high risk of osteoporosis and further fractures.

Of the 223 patients over 50 years, there were 199 patients with PD and 24 patients with either DLB, MSA or PSP (Table 1). Patients over 70 years formed 68% (152/223) of the cohort and, in keeping with the known epidemiology, male patients were more frequent (151/223, 68%). There was almost no difference between the percentage men and women referred for a bone health screen, with 11% of women referred and 9% of men. Of those over 70 years, 66.3% (53/80) were not referred to the GP (Table 2). Interestingly, 80 patients over 70 years had no modifiable risk factors requiring bone health assessment (Supplementary Table 1 contains a breakdown of main results by age). Our audit identified 78 of these 223 patients as having modifiable risk factors, other than age alone, that required bone health assessment according to our recommendations, which were based on the RACGP guidelines [1, 13]. These risk factors included lifestyle factors such as falls/imbalance (*n* = 56), immobility (*n* = 3), fracture history (*n* = 23), drug history including steroids (*n* = 3), and androgen deprivation therapy (*n* = 1). Eight of these patients had both a history of falls and a minimal trauma fracture, all of whom were managed appropriately for osteoporosis. Of these 78 ‘at risk’ patients, 26 already had a diagnosis of osteoporosis and/or were on medication for bone health. Of the remaining 52 ‘at risk’ patients, only 12 (23%) were advised to seek bone health screening with their GP, meaning 40/52 (77%) of these patients did not receive such advice (Table 2). We also used a chi-square test to check for independence between disease duration and referral to their GP. The p-value was 0.34 and, therefore, it is reasonable to accept the null hypothesis at the 5% level. In other words, disease duration and referral to their GP were independent in this study. We also converted each patients Parkinson’s medication to the equivalent levodopa daily dose using published conversion ratios [20] and used chi-square test to check for independence between levodopa equivalent daily dose and referral to GP. As p-value was 0.12 at the 5% level, it is reasonable to assume independence between these two factors in this study.

**Table 1:** A table showing demographic data for patients over 50 included in this audit

Diagnosis	Number	Disease duration^a^ (years) (±SD^b^)	Male	Female	Mean age (years) (±SD^b^)
PD^c^	199	7.7 (5.3)	137	62	71.6 (8.8)
DLB^d^	14	1.8 (1.9)	10	4	77.4 (7.5)
MSA^e^	2	4.5 (0.7)	1	1	69.5 (13.4)
PSP^f^	8	3.7 (3.6)	3	5	73.5 (6.3)
Total^g^	223	7.2 (5.3)	151	72	72 (8.7)

^a^‘Duration’ refers to the mean duration of illness since diagnosis in years

^b^*SD* standard deviation

^c^*PD* Parkinson’s disease

^d^*DLB* Dementia with Lewy bodies

^e^*MSA* multiple system atrophy

^f^*PSP* progressive supranuclear palsy

^g^Total averages were calculated using original values, not mean values from the table

**Table 2:** Management of risk factors for osteoporosis and minimal trauma fracture in patients audited in this clinic

Row number	Risk factor for osteoporosis	Total number	Diagnosis^a^/medication^b^	Referred^c^	Not managed for osteoporosis risk (%^d^)	Recommendation^e^
Age > 50 years *n* = 223						
1	Age over 70 years	152	46	16	90 (59.2)	NA^f^
2	Total risk factors^g^	78	26	12	40 (51.3)	FRAX^h^/DEXA^i^
3	Poor balance or falls	56	18	10	28 (50)	FRAX^h^
4	Immobile	3	2	0	1 (33.3)	FRAX^h^
5	Osteopenia	11	4	1	6 (54.5)	FRAX^h^
6	Previous fracture	23	12	4	7 (30.4)	Fracture history/DEXA^i^/treatment
7	Fractures without falls	15	6	2	7 (46.7)	Fracture history/DEXA^i^/treatment
8	Specific conditions eg. diabetes	30	19	4	7 (23.3)	DEXA^i^
9	Specific medications eg. steroids	4	0	0	4 (100)	DEXA^i^
10	Documented frailty	7	0	6	1 (14%)	Optimisation
11	Exercise recommended?	223	NA^f^	165^j^	58 (26)	Exercise
Age ≤ 50 years *n* = 7						
12	Poor balance or falls	2	0	0	2 (100)	NA^f^
13	Immobile	0	0	0	0 (0)	NA^f^
14	Osteopenia	0	0	0	0 (0)	NA^f^
15	Previous fracture	2	0	0	2 (100)	Fracture history/DEXA^i^/treatment

^a^‘Diagnosis’ here, refers to a diagnosis of osteoporosis

^b^‘Medication’ reflects whether the patient was taking bisphosphonates, denosumab, vitamin D or calcium supplements

^c^‘Referred’ here, implies that the patient has been directed back to their GP for a bone health assessment

^d^The percentages are given out of the total number in that row

^e^‘Recommendation’ here, is as advised by RACGP 2024 guidelines

^f^*NA* Not applicable

^g^‘Total risk factors’ here, include falls/imbalance, immobility, fracture history and drug history. The values in the table should not be used to calculate totals as many patients had multiple overlapping risk factors

^h^*FRAX* fracture risk assessment tool

^i^*DEXA* dual energy x-ray absorptiometry

^j^‘Referred’ here relates to patients who had been referred for physiotherapy or recommended exercise

Significantly, the audit identified 23 patients with a documented history of a minimal trauma fracture who were not on appropriate osteoporosis treatment and only seven of these were referred to their GP for further management. Similarly, out of the seven patients with known osteopenia who were not on medication, only one (1/7, 14%,) was referred to their GP for treatment. Of the seven patients who were identified as frail, almost all (6/7, 86%) were referred to their GP for a bone health screen but only one (1/7, 14%) was referred specifically for vitamin D and calcium optimisation. The majority (165/223, 74%) of all patients over 50 years were recommended to undertake exercise or were specifically referred for physiotherapy (Table 2).

## Discussion

This is the first audit to be conducted on bone health assessment and management in patients with PD and related conditions in Australia. This is also the first audit to review the new guidelines on osteoporosis diagnosis and management released in March 2024 [1]. This audit demonstrated that even in a specialist clinic focusing on Parkinson’s and related diseases, 77% (40/52) of ‘at risk’ patients who did not already have a diagnosis or medication for osteoporosis and who fulfilled the criteria for FRAX screening, or a DEXA scan, were not advised to seek bone health screening with their GP. All patients with a minimal trauma fracture and a history of falls were managed appropriately, suggesting that having both a fracture and recurrent falls increases likelihood of referral for bone health screen compared to either factor alone.

As highlighted above, the introduction of updated guidelines in the period of this audit meant that we used a combination of the 2017 and 2024 guidelines [1, 13] to audit the data and provide recommendations. It seemed inappropriate to only use guidelines that were non-existent at the time when the audited consultations were held. It was also deemed futile to use standards derived from old guidelines when newer and more up to date evidence has been made available. Therefore, we used the level of evidence statements in the 2017 [13] and 2024 [1] guidelines to set a threshold for what we determined as best practice in 2024. Screening patients over 70 years and those with certain conditions or medications were graded D [13] and C [1], respectively, in the 2017 guidelines. This indicated that the evidence to support these recommendations was weak and should be used with caution. The 2024 RACGP revision included no additional data to strengthen this practice guideline. Additionally, if all patients over 70 years were referred to the GP for screening, 69% (58/80) of patients over 70 years with no risk factors for osteoporosis would have been unnecessarily referred to the GP for assessment (Supplementary Table 1). For these reasons, in our recommendations, we have not included being over 70 years sufficient to warrant referral for bone health assessment as a stand-alone factor. Similarly, we decided to limit bone health screening to only patients with uncontrolled chronic conditions listed in the RACGP guidelines [1]. Since this audit aimed to highlight the assessment of fracture risk and osteoporosis by clinicians in this clinic, the discrepancy in management and type of investigations between the two guidelines did not alter the overall impact of this audit.

Patients with minimal trauma fractures have twice the risk of further fractures, long-term morbidity and even premature death [1], and thus the evidence for managing these patients appropriately should be emphasised in patients with PD and related conditions, regardless of age. Additionally, roughly half of initial or recurrent minimal trauma fractures manifest in individuals whose BMD is within the normal or osteopenic range [1]. Therefore, these patients are likely to benefit from a fracture risk algorithm and have been included in our recommendations. Supplementation of vitamin D and/or calcium is likely to be beneficial for patients who are in residential care but has been associated with adverse effects and a small relative reduction in fracture risk in otherwise healthy individuals and non-institutionalised individuals. It is possible that some information regarding a patient’s past medical history may not be available or recalled at a specialist consultation. Thus, recommendations here regarding bone health screening are felt to be best directed by the patient in consultation with their regular GP to avoid duplicating investigations and management. Additionally, we have considered that neurology specialists referring people for DEXA scans may be seen as inappropriate by the government Medicare system. Using this approach, we have created the recommendations below to improve the management of osteoporosis screening, during specialist consultation, within this parkinsonian patient group.

### Specialist recommendations

(i) All patients over 50 years with a history of two or more falls without an external cause in the past 12 months; objective balance deficit on examination; immobility; and/or a history of osteopenia should be directed to have an osteoporosis screen with their GP, ideally using a fracture risk assessment tool such as FRAX (Australia).
(ii) All patients who have sustained a minimal trauma fracture should be directed to the GP for a bone health assessment and review of fracture sites, followed by treatment or DEXA scan depending upon the results of the assessment.
(iii) All patients taking glucocorticoids for $\ge$ 4 months, androgen deprivation therapy or aromatase inhibitors, which are known to contribute to osteoporosis, should be directed to have a bone health assessment with their GP, ideally using a DEXA scan.
(iv) All patients who are frail or institutionalised, should be directed to their GP to have their vitamin D and calcium levels optimised by checking dietary intake and through occasional monitoring of serum vitamin D levels.
(v) Any patient aged over 50 years with a condition associated with an increased risk of osteoporosis (e.g. Type I diabetes, coeliac disease, rheumatoid arthritis) should be directed to have a bone health assessment with their GP, especially if their illness is uncontrolled or poorly managed.
(vi) In keeping with the general advice for patients with parkinsonian conditions, exercise should be encouraged as standard practice regardless of a current osteoporosis diagnosis or bone health medication. This will also help to reduce the risk of osteoporotic fractures.
(vii) Advice regarding osteoporosis risk factors, screening, management, and prevention should be documented.

Whilst we do not know if our results are representative of widespread Australian clinical practice, previous work has shown that osteoporosis is often underdiagnosed and undertreated [21]. Amongst the general population, at least 75% of women and 90% of men with risk factors for osteoporosis are not investigated [22]. Another large study revealed that over 80% of post-menopausal Australian women with a prior osteoporotic fracture did not receive investigation or treatment to mitigate the risk of subsequent fractures [23]. Women are known to be at higher risk of osteoporosis [24] as well as faster progression of PD [25]. Although our audit showed little difference between the percentage of men and women referred for a bone health screen, a larger sample size and access to GP records (to highlight any prior screening) would allow us to evaluate the impact of gender on managing bone health in this population.

It has been demonstrated that PD patients are at higher risk of osteoporosis and osteopenia compared to healthy controls [4]. We suspect that similar issues could apply for other neurodegenerative disorders with a propensity for falls e.g. hereditary cerebellar ataxias [26]. In addition to the increased risk of falls, several pathophysiological mechanisms have been shown to cause decreased BMD and may contribute to this increased risk [3]. Anti-parkinsonian drugs have also been linked to an increased fracture risk and are, therefore, referenced in the UK guidelines as a risk factor [7]. For these reasons, we would propose that PD and atypical parkinsonian syndromes should be incorporated into FRAX scoring as a secondary cause of osteoporosis, and included in the Australian osteoporosis guidelines as one of the conditions that warrant fracture risk assessment, as is done in the UK. Additionally, the UK NOGG guidelines suggest increasing fracture risk by 30% if recurrent falls are present. Although falls are present in the RACGP guidelines, we recommend that recurrent falls should be incorporated into FRAX as a risk factor. Other factors, such as autonomic dysfunction [27], levodopa use and duration of Parkinson’s disease should also be considered for inclusion in FRAX calculation specific to PD.

This study is subject to several criticisms. The audit was reliant on case note documentation, which likely influenced the reliability of our findings. For example, patients with cognitive impairment may not recall details, such as previous falls, investigations, or treatments. The audit criteria for ‘falls’ was defined as more than one fall within the last year, aligning with the RACGP guidelines. However, the frequency of falling recorded in consultation is often unclear and instances were only included if a frequency above the criteria was strongly suggested or clearly stated. Imbalance was defined as any mention of unsteadiness or poor balance, which could be either subjective or objective during examination. To ensure clarity, we have specified in our recommendations that imbalance must be objectively observed on examination. Additionally, if patients were on a ‘drug holiday’ from bisphosphonates, had adequate levels of vitamin D and calcium intake or were already identified from previous screening by their GP or another specialist prior, this may not have been documented. It is possible that patients may have been prescribed bisphosphonates or supplements by another specialty or they may have already had, or been referred for, a bone health screen recently. Access to full patient records, especially their general practice record, would provide more information regarding these factors.

Although this audit was conducted only in one centre, the advice could be extrapolated across other settings such as geriatrics and general neurology. Involving only a single centre in Australia limits this study’s generalisability, therefore, an international audit across neurology and geriatrics should be considered. Health economic analyses should be included in future audits to identify potential challenges that could impede national implementation.

Considering the detrimental impact of poor bone health on morbidity and mortality, our findings have important clinical consequences for these patients. We have highlighted several recommendations to implement before undertaking a second audit round in a year and have presented these to the multidisciplinary team at our clinic to raise awareness. Considering the significant consequences of a missed diagnosis, the guidelines in place to identify individuals at risk and the vulnerability of this cohort, we believe that awareness amongst Neurologists and Geriatricians about osteoporosis screening and prevention should be improved.

## Supplementary Information

Below is the link to the electronic supplementary material.

Supplementary file1 (DOCX 29 KB)

## Data Availability

Data will be made available upon reasonable request.
