# Supplementary material for: An audit on the assessment and management of osteoporosis in a Parkinson’s and related diseases clinic in Australia
Source: J Neurol. 2025 Jan 15;272(2):139. doi: 10.1007/s00415-024-12752-z (PMC11735546; doi:10.1007/s00415-024-12752-z)
Supplement: Supplementary file 1 — Supplementary file1 (DOCX 29 KB) [file 415_2024_12752_MOESM1_ESM.docx]

**An audit on the assessment and management of osteoporosis in a Parkinson’s and related diseases clinic in Australia.**

*Journal of Neurology*

Author list: Nethmi Nuwanji Amarasekera MBBS BSc^1^, Janice Taylor MBBS BAppSci^2^, Christopher Coppin BMed MD MMed^2^, Simon J G Lewis MBBCh BSc FRACP FRCP MD^2^,

1 – Imperial College School of Medicine, Imperial College London, London, United Kingdom.

2 - Parkinson’s Disease Research Clinic, Macquarie Medical School, Macquarie University, Sydney, Australia.

Corresponding Author Details

Prof Simon J.G. Lewis, +61 2 9850 2435, simon.lewis@mq.edu.au

Parkinson’s Disease Research Clinic, Macquarie Medical School, Macquarie University, Sydney, Australia, NSW 2109

Supplementary Table 1: Management of risk factors for osteoporosis and minimal trauma fracture in patients audited at this clinic, broken down by age group.

| Row Number | Risk Factor | Total | Diagnosis_^[[1]](#footnote-1)^_/  Medication_^[[2]](#footnote-2)^_ | Referred_^[[3]](#footnote-3)^_ | Not managed (%_^[[4]](#footnote-4)^_) | Recommendation_^[[5]](#footnote-5)^_ |
| --- | --- | --- | --- | --- | --- | --- |
| *Age ≥ 70 years n=152* | | | | | | |
| 1 | Over 70 years | 152 | 46 | 16 | 90 (59.2) | NA_^[[6]](#footnote-6)^_ |
| 2 | Poor balance/falls | 36 | 13 | 6 | 17 (47.2) | FRAX_^[[7]](#footnote-7)^_ |
| 3 | Immobile | 2 | 2 | 0 | 0 (0) | FRAX_24_ |
| 4 | Osteopenia | 9 | 3 | 1 | 5 (55.6) | FRAX_24_ |
| 5 | No risk factors | 80 | 22 | 5 | 53 (66.3) | NA_23_ |
| *50 < Age ≤ 70 years n=71* | | | | | | |
| 6 | Poor balance/falls | 20 | 5 | 4 | 11 (55) | FRAX_24_ |
| 7 | Immobile | 1 | 0 | 0 | 1 (100) | FRAX_24_ |
| 8 | Osteopenia | 2 | 1 | 0 | 1 (50) | FRAX_24_ |
| *Age > 50 years n=223* | | | | | | |
| 9 | Poor balance/falls | 56 | 18 | 10 | 28 (50) | FRAX_24_ |
| 10 | Immobile | 3 | 2 | 0 | 1 (33.3) | FRAX_24_ |
| 11 | Osteopenia | 11 | 4 | 1 | 6 (54.5) | FRAX_24_ |
| 12 | Fractures | 23 | 12 | 4 | 7 (30.4) | Fracture history/ DEXA_^[[8]](#footnote-8)^_/Treatment |
| 13 | Fractures w/o falls | 15 | 6 | 2 | 7 (46.7) | Fracture history/ DEXA_25_/Treatment |
| 14 | Total risk factors_^[[9]](#footnote-9)^_ | 78 | 26 | 12 | 40 (51.3) | FRAX_24_/ DEXA_25_ |
| 15 | Specific conditions | 30 | 19 | 4 | 7 (23.3) | DEXA_25_ |
| 16 | Specific medications | 4 | 0 | 0 | 4 (100) | DEXA_25_ |
| 17 | Frailty | 7 | 0 | 6 | 1 (14%) | OPTIMISATION |
| 18 | Exercise recommended | 223 | NA^23^ | 165^[[10]](#footnote-10)^ | 58 (26) | Exercise |
| *Age ≤ 50 years n=7* | | | | | | |
| 19 | Poor balance/falls | 2 | 0 | 0 | 2 (100) | NA^23^ |
| 20 | Immobile | 0 | 0 | 0 | 0 (0) | NA^23^ |
| 2§ | Osteopenia | 0 | 0 | 0 | 0 (0) | NA^23^ |
| 22 | Fractures | 2 | 0 | 0 | 2 (100) | Fracture history/ DEXA^25^/Treatment |

1. ‘Diagnosis’ refers to a diagnosis of osteoporosis. [↑](#footnote-ref-1)
2. ‘Medication’ suggests the patient was taking bisphosphonates, denosumab, vitamin D or calcium supplements. [↑](#footnote-ref-2)
3. ‘Referred’ implies that the patient has been directed back to their GP for a bone health assessment, [↑](#footnote-ref-3)
4. The percentages are given out of the total number in that row. [↑](#footnote-ref-4)
5. ‘’Recommendation’ is as advised by RACGP 2024 guidelines. [↑](#footnote-ref-5)
6. NA= not applicable. [↑](#footnote-ref-6)
7. FRAX = fracture risk assessment tool. [↑](#footnote-ref-7)
8. DEXA= dual energy x-ray absorptiometry. [↑](#footnote-ref-8)
9. ‘Total risk factors’ include falls/imbalance, immobility, fracture history and drug history. The values in the table should not be used to calculate totals as many patients had multiple overlapping risk factors. [↑](#footnote-ref-9)
10. ‘Referred’ here, relates to patients who had been referred for physiotherapy or recommended exercise. [↑](#footnote-ref-10)
